# Current Status and Development Trend of Research on Polymer-Based Kinetic Inhibitors for Natural Gas Hydrates

**DOI:** 10.3390/polym16141985

**Published:** 2024-07-11

**Authors:** Shujie Liu, Sunan Wang, Jiansheng Luo, Yilong Xu, Liangliang Ren, Xiong Xiang, Tie Geng, Botao Xu, Lei Guo

**Affiliations:** 1 CNOOC Hainan Energy Co., Ltd., Haikou 570105, China; liushj@cnooc.com.cn (S.L.); xuyl2@cnooc.com.cn (Y.X.); 2 China Oilfield Services Limited, Sanhe 065201, China; luojsh@cosl.com.cn (J.L.); renll4@cosl.com.cn (L.R.); xiangxiong@cosl.com.cn (X.X.); gengtie@cosl.com.cn (T.G.); xubt@cosl.com.cn (B.X.); guolei5@cosl.com.cn (L.G.)

**Keywords:** natural gas hydrates, kinetic inhibitors, research history, structure composition, mechanisms of inhibition

## Abstract

As the understanding of natural gas hydrates as a vast potential resource deepens, their importance as a future clean energy source becomes increasingly evident. However, natural gas hydrates trend towards secondary generation during extraction and transportation, leading to safety issues such as pipeline blockages. Consequently, developing new and efficient natural gas hydrate inhibitors has become a focal point in hydrate research. Kinetic hydrate inhibitors (KHIs) offer an effective solution by disrupting the nucleation and growth processes of hydrates without altering their thermodynamic equilibrium conditions. This paper systematically reviews the latest research progress and development trends in KHIs for natural gas hydrates, covering their development history, classification, and inhibition mechanisms. It particularly focuses on the chemical properties, inhibition effects, and mechanisms of polymer inhibitors such as polyvinylpyrrolidone (PVP) and polyvinylcaprolactam (PVCap). Studies indicate that these polymer inhibitors provide an economical and efficient solution due to their low dosage and environmental friendliness. Additionally, this paper explores the environmental impact and biodegradability of these inhibitors, offering guidance for future research, including the development, optimization, and environmental assessment of new inhibitors. Through a comprehensive analysis of existing research, this work aims to provide a theoretical foundation and technical reference for the commercial development of natural gas hydrates, promoting their safe and efficient use as a clean energy resource.

## 1. Introduction

**Figure 1:**
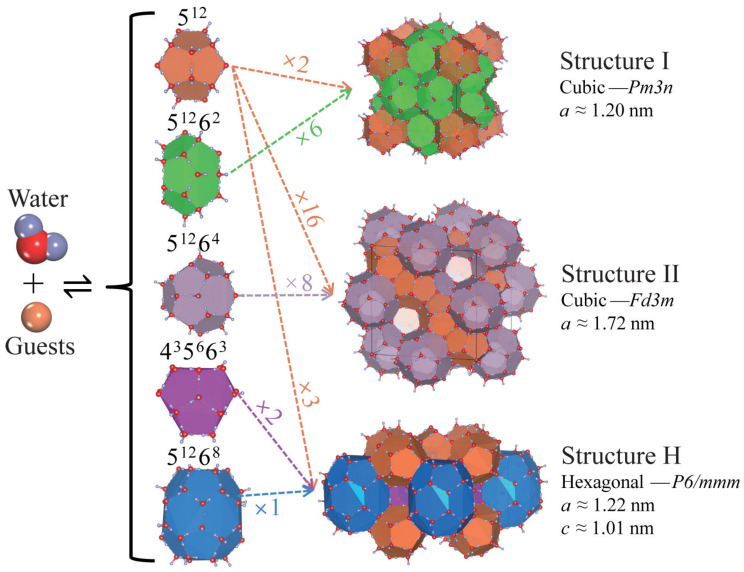
Cages and three common crystal structures of natural gas hydrates (NGHs) [3,11].

Natural gas hydrates are ice-like crystalline compounds formed by guest molecules, such as methane, and host framework cages, as depicted in Figure 1 [1,2,3]. These hydrates are widely distributed in nature, primarily in marine sediment deposits. Global sediment deposits are estimated to contain vast amounts of natural gas hydrates, with reserves more than twice that of conventional natural gas, presenting substantial development potential. Furthermore, natural gas hydrates are considered an environmentally friendly clean energy source because their combustion produces significantly lower carbon dioxide emissions compared to fossil fuels such as coal and oil. Thus, natural gas hydrates are regarded as a new clean energy resource of the 21st century with immense commercial development potential [4,5,6,7,8,9,10].

**Figure 2:**
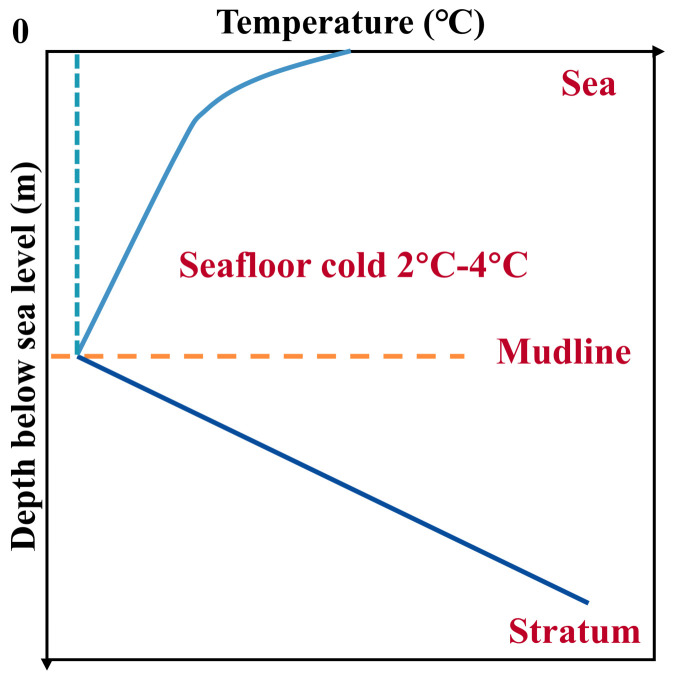
Schematic diagram of ambient temperature change in deep water.

**Figure 3:**
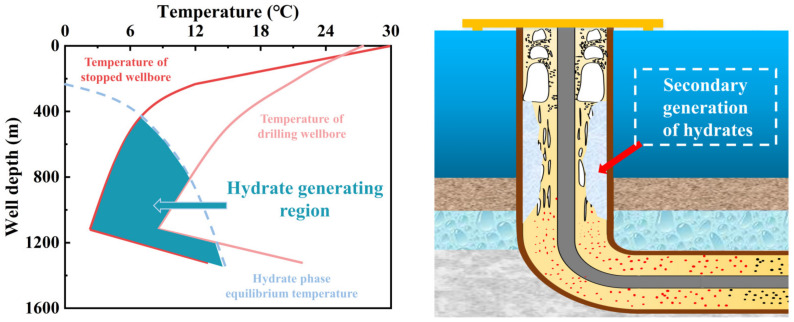
Schematic diagram of secondary hydrate generation in the wellbore during the deepwater drilling process.

In recent years, the extraction of oil resources has gradually expanded to deep-sea hydrate layers, presenting increasing technical challenges for drilling operations [12,13]. The unique low-temperature, high-pressure environment of shallow deep-water drilling (as shown in Figure 2) easily induces the secondary formation of natural gas hydrates in the wellbore’s annular drilling fluid. These hydrates can block various pipelines and blowout preventers, leading to potential safety incidents, as illustrated in Figure 3 [14,15]. Additionally, hydrates crystallize on the seabed, occupying sediment pores, reducing sediment volume, and lowering drilling efficiency [16,17]. Therefore, effectively inhibiting the secondary formation of hydrates has become crucial for improving drilling efficiency and safety [18].

Natural gas hydrate inhibitors are essential for hydrate drilling. They are categorized into thermodynamic inhibitors and kinetic inhibitors [19]. Thermodynamic inhibitors, such as NaCl, KCl, CaCl_2_, and ethylene glycol, alter the phase equilibrium curve of hydrates [20,21]. However, these inhibitors require large quantities and are not environmentally friendly. In contrast, kinetic hydrate inhibitors (KHIs), such as PVP, PVCap, and VC-713, do not change the thermodynamic equilibrium conditions of hydrate formation and instead inhibit hydrate formation by affecting the nucleation and growth processes [22,23,24,25].

Compared to thermodynamic inhibitors, KHIs are considered a more attractive option due to their lower dosage requirements and environmental friendliness. KHIs provide a more efficient and economical solution, effectively delaying hydrate formation without the need for large quantities of chemicals. This significantly reduces treatment costs and potential hazards to the drilling environment. Additionally, the flexibility of KHIs is a notable advantage. They can act in the early stages of hydrate formation, effectively controlling the growth rate and final crystal size by disrupting the nucleation process of the hydrate’s microstructure [26,27,28]. This mechanism relies on a deep understanding of the molecular structure and action mechanisms of KHIs, enabling researchers to design more efficient inhibitors to address the complex issues of secondary hydrate formation.

This paper delves into the current research status of kinetic inhibitors for natural gas hydrates and focuses on their development trends. By comprehensively analyzing the significant progress made in this field over the years, it proposes future research directions and relevant design concepts for kinetic inhibitors.

## 2. Natural Gas Hydrate Kinetic Inhibitors Research Development History

Over the past thirty years, research on KHIs has garnered widespread attention [29,30,31,32]. Compared to thermodynamic inhibitors, KHIs are favored for their lower dosage, reduced cost, environmental friendliness, and high inhibition efficiency. KHIs are primarily water-soluble polymers that delay nucleation by slowing or preventing the growth of hydrate crystals [33]. They adsorb onto the surface of hydrate particles and form hydrogen bonds, thereby delaying crystal growth. Small amounts of KHIs can alter the growth characteristics of structure II hydrates, while for structure I hydrates, they trigger rapid branching phenomena [34,35,36]. In summary, due to their low dosage, reduced cost, and environmental friendliness, KHIs have become an effective means of preventing and delaying hydrate formation. They significantly impact the nucleation and growth processes of hydrates, particularly in altering growth characteristics and controlling crystal branching.

**Figure 4:**
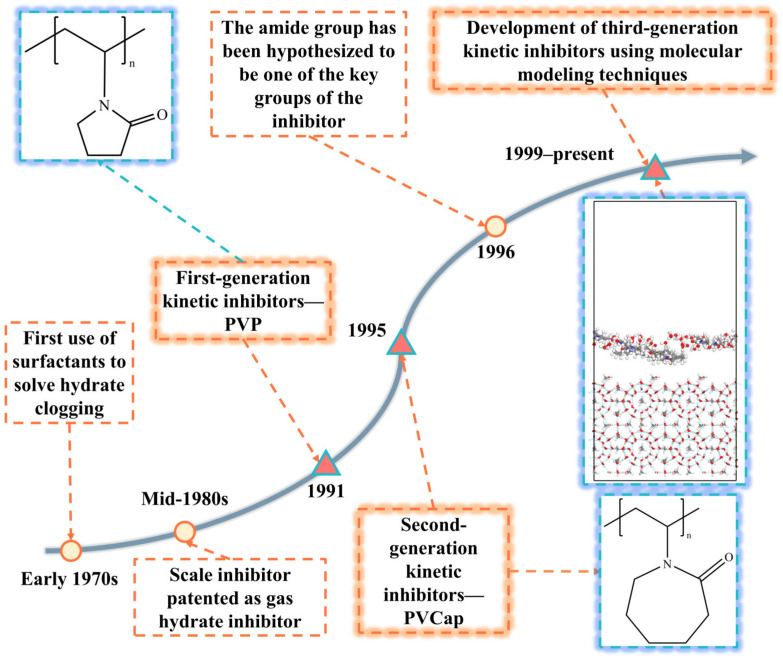
History of kinetic hydrate inhibitor research.

Common kinetic inhibitors include polyvinylpyrrolidone (PVP) and polyvinylcaprolactam (PVCap) [37,38]. As shown in Table 1 and Figure 4, research on kinetic inhibitors can be divided into three stages: the first generation (1991–1995), the second generation (1995–1999), and the third generation (1999 to present). The first-generation kinetic inhibitors, primarily PVP, were selected through experimental evaluation [39]. The second-generation inhibitors were improved based on the molecular structure of PVP through structure–activity relationship analysis and molecular design, with representative compounds including PVCap [40]. The third-generation inhibitors have been developed through computer molecular simulation and design techniques, resulting in new compounds with better inhibition effects [41]. Research and development in this field have progressed from basic screening to molecular design, continuously advancing and innovating to develop more efficient and environmentally friendly inhibitors to control hydrate formation.

### 2.1. First-Generation Kinetic Inhibitors

Since 1991, research on kinetic inhibitors has progressively deepened. Initially, researchers utilized alkyl aryl sulfonates and their salts as kinetic inhibitors [42]. In 1993, researchers proposed using tyrosine and its derivatives, along with vinyl-containing polymers and complexing agents, as hydrate inhibitors [43]. In 1994, polyvinylpyrrolidone (PVP) was discovered to delay hydrate formation. In the same year, hydroxyethyl cellulose (HEC) demonstrated similar effects, but PVP proved more effective [44]. By 1995, experiments confirmed several effective chemical additives, including PVP, BASF F-127, Mirawet ASC, Surfynol-465, sodium dodecyl sulfate (SDS), Mirataine CBS + PVP, and SDS + PVP [24]. Among these inhibitors, PVP is the most representative, with a relative molecular mass ranging from 10,000 to 350,000. Its monomer structure contains a five-membered lactam ring [45,46].

The effectiveness of PVP lies in its structural characteristics, particularly the molecular weight range and the monomer structure of the five-membered lactam ring. It inhibits hydrate growth through hydrogen bonding and van der Waals forces, a mechanism validated by molecular simulations [7]. Consequently, PVP has become a research focus due to its structural properties and its ability to prevent hydrate growth. This series of studies [6,7,8,9,10,47,48,49,50,51,52], by exploring the effects, testing, and molecular mechanisms of chemical additives, has revealed the potential and mechanisms of PVP and other inhibitors in preventing hydrate formation, representing progress and innovation in the field of hydrate formation control.

### 2.2. Second-Generation Kinetic Inhibitors

**Figure 5:**
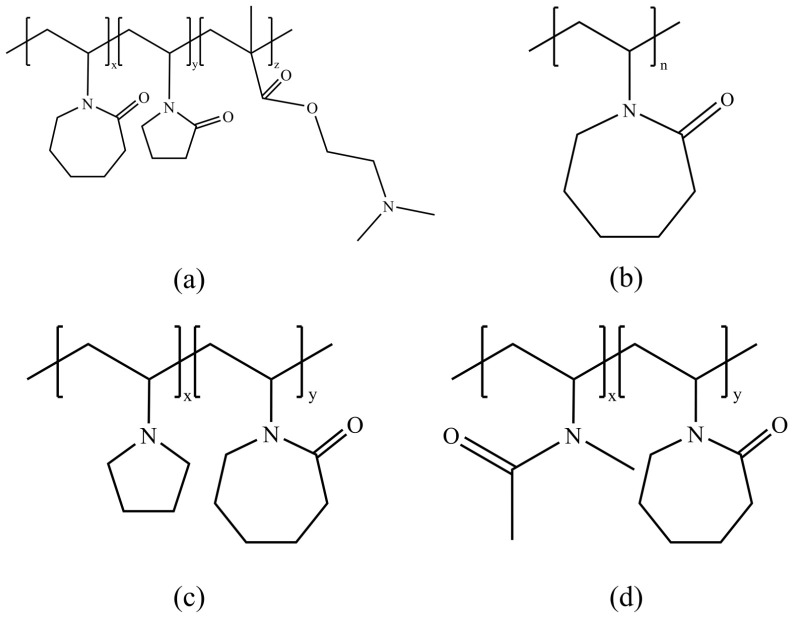
Second-generation kinetic inhibitor structure: (**a**) VC-713, (**b**) PVCap, (**c**) Poly(VP-VC), and (**d**) VIMA-VCap.

Although effective at relatively high temperatures, PVP performance under supercooling conditions around 5 °C is limited and may even sometimes promote hydrate formation, highlighting the urgency of developing more effective KHIs [53]. The Gaffix VC-713 polymer, a terpolymer consisting of vinylcaprolactam (VCap), vinylpyrrolidone (VP), and dimethylaminoethyl methacrylate (DMAEMA), exhibits superior performance compared to PVP [54], as shown in Figure 5a. Specifically, the addition of PVCap or Gaffix VC-713 can significantly delay the nucleation process of hydrates. The structure of PVCap [55], shown in Figure 5b, and its copolymer with VP and VCap, Poly(VP-VC), shown in Figure 5c, both exhibit good inhibition effects [56]. Additionally, other polymers such as polyelectrolytes, polyethers, and polyvinylamines, used as co-inhibitors with PVCap, can further enhance performance through synergistic effects between the polymers. The VIMA-VCap copolymer, synthesized by RF Company, consists of N-methyl-N-vinylacetamide and VCap in a 1:1 ratio and outperforms PVCap [57], with a supercooling enhancement of 2 to 3 °C in sapphire cell tests. RF Company also synthesized and tested other VCap copolymers, including those with vinyl imidazole, all of which showed good effects. The structure of the VIMA-VCap copolymer is shown in Figure 5d.

The inhibitory effects of PVCap, PVP, VC-713, and VP-VCap copolymers are closely related to system pressure, salinity, and inhibitor concentration and are influenced by multiple factors [58]. Experiments show that the inhibitory effect of the VP-VCap copolymer (molar ratio 25/75) is comparable to that of VC-713 or PVCap, confirming the copolymer’s effectiveness [59]. Methanol and low-salinity negatively affect PVCap’s performance, while high salinity (>5.5%) is beneficial, highlighting the significant role of salinity in inhibiting hydrate formation [60]. The relative molecular mass of PVCap significantly impacts its performance, particularly when the molecular mass is 900 [61], demonstrating optimal supercooling and emphasizing the importance of molecular mass in inhibitor performance. PVCap’s interaction with hydrate solutions is more effective as a kinetic inhibitor compared to PVP. The performance of PVP can be enhanced through chemical modification by adding hydroxyl groups [62], demonstrating the potential for optimizing inhibitor performance [63]. VC-713 also exhibits good interaction with hydrate solutions. Low-dose inhibitors (PVP, PVCap, and VC-713) significantly extend hydrate decomposition time, with PVCap showing the most pronounced effect, highlighting its superiority in inhibition [64]. These findings are crucial for designing and selecting more effective hydrate inhibition methods, particularly noting PVCap’s superior performance in high salinity and the potential to enhance PVP performance through chemical modification.

PVCap are significant differences in inhibiting Type II and Type I hydrate structures [55]. The supercooling of Type I hydrates is lower than that of Type II hydrates, which is closely related to the high symmetry of Type I hydrate crystals [65,66,67,68,69]. Experiments have shown that under dynamic stirring conditions, compared to static solutions, the growth of hydrate crystals can be inhibited with a lower dose of KHIs, indicating higher inhibition efficiency under dynamic conditions [70]. During shutdown periods, the required inhibitor dose under static conditions may be higher than under flowing conditions, which is crucial for designing hydrate inhibition strategies during shutdowns. Additionally, compared to high-molecular-weight polymers, low-molecular-weight polymers perform better under static conditions due to their faster diffusion to the hydrate surface. Therefore, the performance of PVCap is influenced by the type of hydrate structure, the stirring state of the solution, and the molecular weight of the polymer [40].

**Figure 6:**
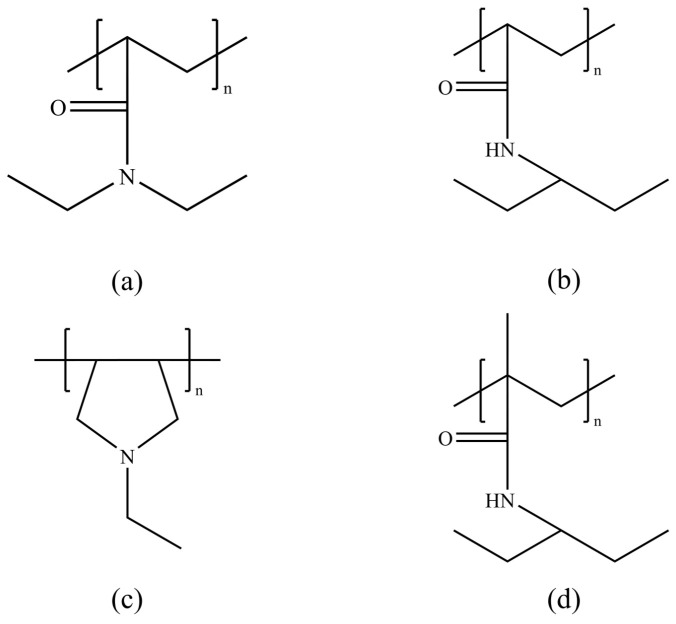
Second-generation kinetic inhibitor structures: (**a**) polydiethylacrylamide, (**b**) polyisopropylacrylamide, (**c**) polymaleimide, and (**d**) polyisopropylmethacrylamide.

Exxon’s research indicates that the amide group plays a crucial role in KHI polymers [71,72]. It promotes hydrate cavity formation through its connection with hydrophobic groups and inhibits hydrate nucleation by forming hydrogen bonds between the hydroxyl oxygen atom of the amide group and water molecules [73]. Among various amide-containing polymers, including poly(diethylacrylamide), poly(isopropylacrylamide) (as shown in Figure 6a,b), polyvinylamine, polypropylamine, and polymaleimide (as shown in Figure 6c), acrylamide polymers exhibit the best KHI effects [74]. Notably, poly(acryloylpyrrolidone), poly(diethylacrylamide), and poly(isopropylacrylamide) show the most outstanding performance [75]. The research also points out that adding a methyl group to the main chain of acrylamide polymers can further enhance their performance [76]. For instance, poly(isopropylmethacrylamide) (as shown in Figure 6d) has a supercooling degree 2 °C higher than that of poly(isopropylacrylamide) [77]. This finding emphasizes the potential for improving polymer KHI performance through specific configurations of functional groups and optimization of chemical structures. Particularly for amide-containing acrylamide polymers, this indicates their high effectiveness in inhibiting hydrate nucleation. This research provides important guidance for designing and developing new, efficient KHIs, highlighting the importance of considering specific functional group roles and interactions in chemical structure design.

**Figure 7:**
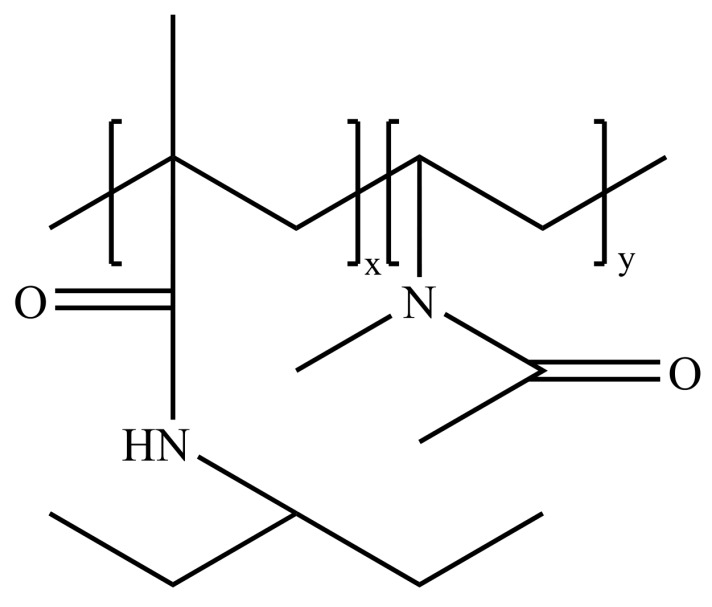
VIMA-IPMA structure.

High-performance polyIPMA, particularly the type synthesized with butyl ethylene glycol, demonstrates significant competitive advantages over PVCap [32]. These advantages are evident not only in its basic performance but also in the superior performance of hydroxy-terminated polyIPMA and polyIPMA with a bimodal molecular weight distribution in inhibiting hydrate formation compared to conventional polyIPMA [78,79]. Experiments have shown that as little as 0.5% of bimodal molecular weight distribution polyIPMA can significantly delay hydrate formation, achieving the highest supercooling performance to date (24.1 °C supercooling, lasting up to 20 h). The performance enhancement is closely related to the molecular weight, size, and activity of the samples. Notably, high-molecular-weight samples are more efficient in preventing hydrate nucleation and growth. Meanwhile, polyVIMA, a polyvinylamine polymer, has shown only weak kinetic inhibition properties. However, copolymers of VIMA with other alkylamine polymers, similar to the VIMA-VCap copolymer shown in Figure 7, exhibit high supercooling performance, providing a new approach to enhancing hydrate inhibitor performance. In summary, specifically synthesized optimized polyIPMA polymers, such as the hydroxy-terminated version synthesized with butyl ethylene glycol and the version with a bimodal molecular weight distribution, have significant performance advantages in kinetically inhibiting hydrate formation. This emphasizes the potential of optimizing KHI performance by adjusting synthesis methods and molecular weight distribution. Additionally, the application of VIMA copolymers with other alkylamine polymers opens new pathways for improving the overall performance of hydrate inhibitors.

The VIMA-vinyl butyrate copolymer exhibited excellent anti-nucleation and crystal growth inhibition capabilities in high-pressure, small-loop experiments [80], outperforming PVCap, polyIPMAM, and polyAP and demonstrating its potential as a hydrate inhibitor. The butyrate group is considered a key factor, particularly effective in the VIMA-vinyl butyrate copolymer. Although the specific mechanism of interaction between ester and amine groups with the hydrate surface is not yet clear, it is hypothesized that hydrogen bonds formed by ester groups may be weaker than those formed by amine groups. In practical applications, polyvinylamide combined with quaternary compounds (such as TBAB) has performed well as a hydrate inhibitor (THI) and has been successfully applied in pipelines [81]. Specifically, the combination of TPAB or TBAB with PVCap enhances the inhibitory effect, and molecular simulation studies indicate that TPAB can be embedded into the hydrate crystal surface, effectively preventing its growth [59,82,83]. The KHI performance of polyAP is superior to Gaffix VC-713, especially when its molecular weight is in the range of 1000 to 3000. Although PVCap and PVP can be used as co-inhibitors with polyAP to enhance its performance, cost issues limit the widespread application of polyAP. Overall, the VIMA-vinyl butyrate copolymer stands out for its excellent hydrate inhibition performance, particularly due to its butyrate group, providing valuable direction for developing new, efficient hydrate inhibitors. The successful application of polyvinylamide and quaternary compound combinations has shown significant effects in hydrate inhibition, while polyAP and its combination with other polymers has shown potential for improving hydrate inhibition effects.

### 2.3. Third-Generation Kinetic Inhibitors

Molecular simulation technology is a vital tool for designing more effective low-dosage hydrate inhibitors (LDHIs), including KHIs and anti-agglomerants (AAs) [84]. This technology can identify functional groups that strongly interact with hydrate structure surfaces and integrate these groups into water-soluble polymers to create KHIs or embed them into surfactants to create AAs [85]. RF Company’s molecular simulation studies have specifically found that alkylamine compounds containing three to four carbon atoms, particularly when the alkyl groups are branched structures like isopropyl and isobutyl, exhibit the strongest interactions with Type II hydrate surfaces, showing excellent inhibitory potential [86]. Using polyisopropylacrylamides (polyIPA) as an example, this instance confirms the reliability of the molecular simulation results, although its inhibition effect is slightly lower than that of Gaffix VC-713 in actual tests.

Patent W001/77270 and Shell’s patent CN1685130A propose an innovative method using dendritic compounds as hydrate inhibitors [87]. These dendritic compounds are three-dimensional, multi-branched oligomeric or polymeric molecules that include a core, multiple branching levels, and outer end groups. By combining at least one dendritic compound with a molecular mass of at least 1000 and at least one small molecular substance with a molecular mass of less than 1000 (such as polyalkylene imine, polyallylamine, etc.), along with a surfactant, a mixture capable of effectively inhibiting hydrate formation is formed. This mixture achieves synergistic control of hydrate formation through the inhibition of hydrate nucleation or crystal growth by the macromolecular and small molecular components and the surfactant’s function as a solvent or surface tension regulator. The small molecular substances can be further modified to include non-cyclic or cyclic side chain groups with heteroatoms such as N, O, or S. The surfactant can be cationic, anionic, or nonionic, such as polyoxyethylene ether or sorbitan. This method showcases an efficient hydrate inhibition strategy through the synergistic effect of the specific structural characteristics of the dendritic compounds, small molecular substances, and surfactants, effectively inhibiting the formation and growth of hydrates.

Dendritic compounds with high molecular weights, particularly polyamide ester types, demonstrate excellent performance in hydrate inhibition due to their unique non-cyclic or cyclic side chain groups containing N, O, or S heteroatoms [88]. Notable examples include ASTRAMOL poly(propylene imine) dendrimers and HYBRANE hyperbranched polyamide esters. The main chains of these compounds, formed by the reaction of cyclic anhydrides with alkanol amines, feature hydrophobic amine groups similar to early kinetic inhibitors like PVCap and polyIPMA. The hydrophobic groups interact with the hydrate surface via van der Waals forces, while the amine groups form hydrogen bonds with water molecules, effectively preventing hydrate particle growth [89]. Due to their highly branched structures, even polyamides with a molecular weight of around 1500 exhibit good KHI performance and bind more closely to the hydrate surface [90]. Research by Baker Petrolite indicates that polyamide ester KHIs perform better in handling Type I hydrate structures formed under high methane content [91]. Two field trials and subsequent field applications have demonstrated the effectiveness and application potential of these polymers in specific oil and gas field conditions. Overall, dendritic compounds with high molecular weights, especially polyamide esters, enhance interactions with hydrate surfaces due to their branched structures with specific side chain groups, showing particular suitability and effectiveness in inhibiting hydrate formation. These advances not only promote further research on hydrate inhibitors but also drive their field applications, particularly in handling high methane content Type I hydrate structures.

**Figure 8:**
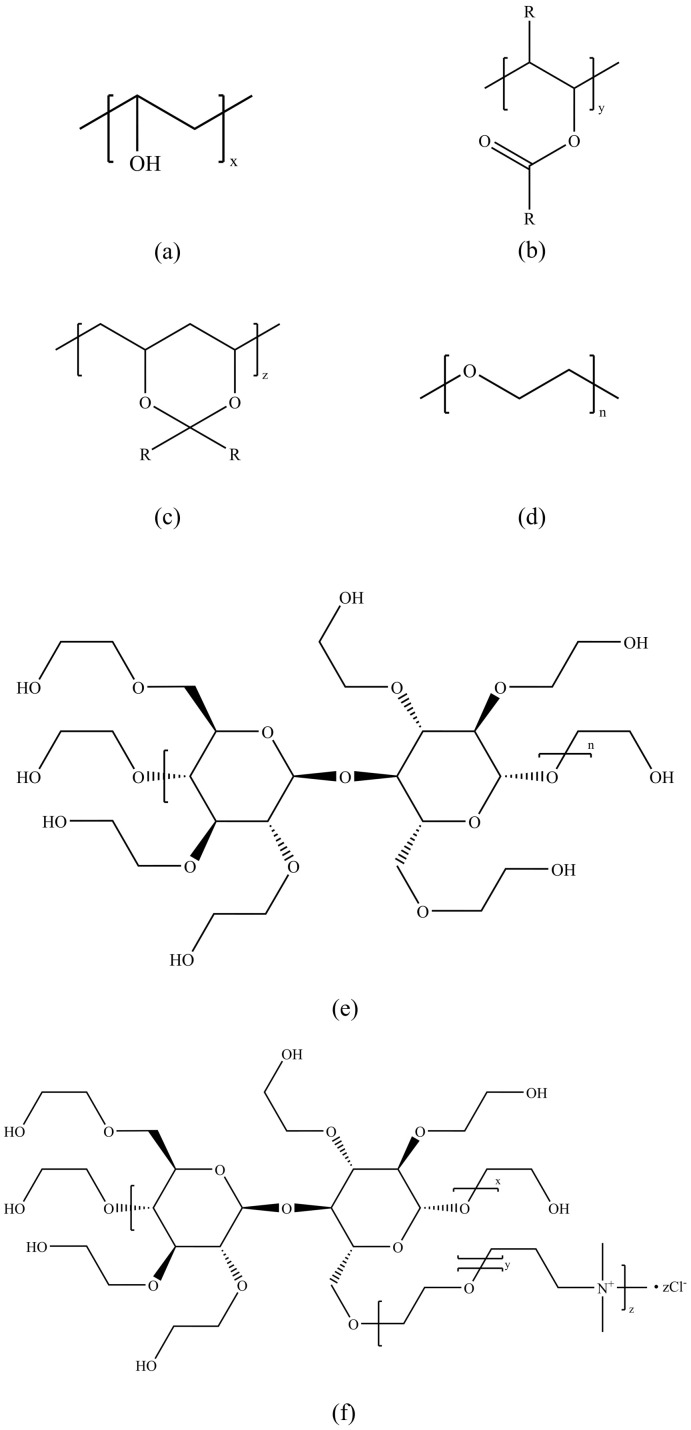
Third-generation kinetic hydrate inhibitor structures: (**a**) polyvinyl alcohol i, (**b**) polyvinyl alcohol ii, (**c**) polyvinyl alcohol III, (**d**) PEO, (**e**) HEC, and (**f**) HECE.

The patent filed by Kuraray Specialties Europe in 2002 demonstrates the effectiveness of polymers based on polyvinyl alcohol and its aldehyde reaction derivatives as KHIs [32], as shown in Figure 8a–c, with particular emphasis on polymers containing acetal ester functional groups, where butyraldehyde is preferred. These polymers are suitable not only as KHIs but also as anti-agglomerants (AAs). Additionally, polyethylene oxide (PEO) [92], as shown in Figure 8d, has been proven to be an effective methane hydrate inhibitor, capable of quickly preventing hydrate formation at very low concentrations (0.1%) and significantly reducing the formation rate thereafter, outperforming pure water. Although hydroxyethyl cellulose (HEC), shown in Figure 8e, and modified hydroxyethyl cellulose (HECE), shown in Figure 8f, can slow down the hydrate formation rate [93], their effects are weaker compared to polyvinylpyrrolidone (PVP). Particularly at a concentration of 1%, the inhibitory performance of PEO is more pronounced. In summary, polymers derived from polyvinyl alcohol and aldehyde reactions, especially those containing acetal ester functional groups, have been proven effective as hydrate inhibitors. PEO shows excellent inhibitory effects as a methane hydrate inhibitor at very low concentrations, while HEC and HECE, despite having inhibitory effects, are weaker in comparison.

**Figure 9:**
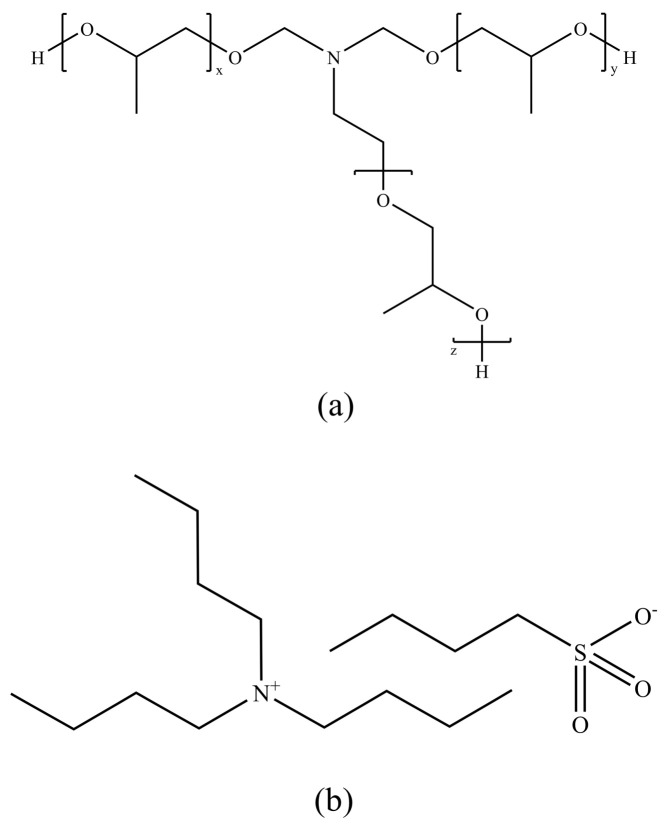
Third-generation kinetic hydrate inhibitor structures: (**a**) polyalkylamine oxides and (**b**) TBAPS.

In 2003, Akzo Nobel Company proposed a patent introducing a novel low-dosage hydrate inhibitor (LDHI) based on polyalkylene oxide amine [94], marking a significant advancement in hydrate control technology. The production process of this inhibitor prefers alkylation with propylene oxide (PO), with triethanolamine as the amine of choice, although ammonia and other alkanol amines are also suitable. The patent specifically mentions the quaternary ammonium form of the amine, which shows promising potential in enhancing the inhibitory effect, particularly the quaternized product of triethanolamine containing 14.9 PO units (Figure 9a), demonstrating the most significant effect. Experimental data indicate that without the addition of polyalkylene oxide amine, the initial temperature for hydrate formation is 5.6 °C. However, with the addition of 1.0% polyalkylene oxide amine, the hydrate formation temperature significantly drops to 0.5 °C [95]. This demonstrates the effectiveness of polyalkylene oxide amine in reducing the hydrate formation temperature, and further enhancement of its performance as an LDHI can be achieved through quaternization and fine-tuning the number of PO units in the polymer structure.

Storr et al.’s research combined molecular simulation and experimental testing to investigate a zwitterionic kinetic hydrate inhibitor (KHI), namely tributylammonium propyl sulfonate (TBAPS) [36,96,97], as shown in Figure 9b. They found that TBAPS is slightly more effective than polyvinylpyrrolidone (PVP) in inhibiting hydrate formation. Its unique mechanism of action involves covering rather than directly adhering to the hydrate surface cavities. This mechanistic difference opens up new perspectives for the design and application of hydrate inhibitors. However, molecular simulations in the design phase did not account for interactions between free water and low-dosage hydrate inhibitors (LDHIs), potentially limiting the comprehensive understanding of inhibitor performance. Despite this limitation, molecular simulation technology successfully designed a new product, J3, which significantly outperforms PVCap on the market, particularly in extending the induction period of Type II hydrates, achieving a remarkable effect of over 4000 min [98]. This achievement not only demonstrates the potential of molecular simulation in guiding the development of new hydrate inhibitors but also highlights the application potential of new inhibitors like J3 in enhancing hydrate management efficiency and safety.

In his 2004 study, Huang [99] investigated the effectiveness of antifreeze proteins (AFPs) as KHIs. He found that AFPs effectively inhibit hydrate formation by lowering the freezing point of aqueous solutions through strong interactions [100,101,102,103,104,105,106,107,108]. This process extends the induction time for hydrate formation, slows the conversion rate to methane hydrates, and prevents the reformation of hydrates. This discovery offers a potential method for using natural inhibitors to prevent hydrate formation. Meanwhile, BASF’s patent proposed an innovative approach using graft polymers as gas hydrate inhibitors. These graft polymers, which can be water-soluble or water-dispersible, are optimized by selecting different polymer bases and grafting monomers. Their design combines hydrophilic and hydrophobic components, along with potential biodegradability, to enhance gas hydrate inhibition efficiency and environmental friendliness.

## 3. Structural Composition Classification of Natural Gas Hydrate Kinetic Inhibitors

**Figure 10:**
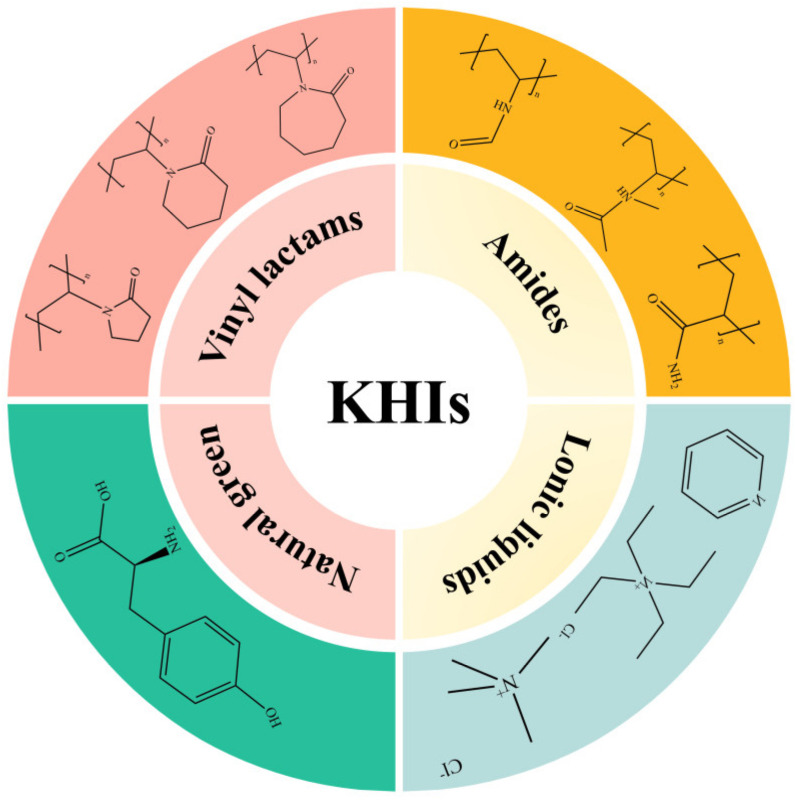
Category of kinetic inhibitors.

Since their discovery, KHIs have become a central focus in hydrate research. In the early exploratory phase, foundational research was conducted that paved the way for the development of kinetic hydrate inhibitors (KHIs). During this time, researchers identified the potential of various chemical compounds to inhibit hydrate formation, leading to the discovery of fundamental principles and mechanisms that would later be harnessed in more advanced inhibitor formulations. Beginning in the 1970s, researchers have extensively screened various reagents and successfully identified many effective KHIs [109]. Classification refers to the systematic arrangement of KHIs into defined groups based on shared chemical characteristics and mechanisms of action. In contrast, categorization involves grouping KHIs based on their functional performance and specific applications in hydrate inhibition. This distinction is crucial for understanding both the chemical properties of the inhibitors and their practical utility in various operational contexts. These KHIs can be broadly categorized into four types—vinyl lactam polymers, amide polymers, natural green inhibitors, and ionic liquids—based on their structure, as shown in Figure 10.

### 3.1. Vinyl Lactam Polymer Inhibitors

Vinyl lactam polymers, such as PVP (polyvinylpyrrolidone), PVPip (polyvinyl piperidine) [110], PVCap (polyvinylcaprolactam), and PVACO (polyvinylazocyclooctanone) [111], exhibit significant potential in hydrate inhibition due to their lactam ring structures. Research indicates that increasing the size of the lactam ring can effectively enhance inhibition performance [112,113]. PVP, the earliest discovered polymer with inhibitory effects, represents the first generation of kinetic inhibitors [114]. Institutions like the Colorado School of Mines have conducted in-depth studies on these compounds, finding that synthesizing new vinyl lactam polymers and their copolymers (such as PVCap) can achieve better inhibition performance than PVP [70,115,116,117]. Experiments indicate that copolymerizing vinyl lactam compounds with other compounds, such as 1-vinyl-3-alkylimidazolium bromide copolymers or terminal hydroxyl poly(N-vinyl caprolactam), can produce new inhibitors with both excellent hydrate inhibition performance and high solubility. These results confirm that the hydrate inhibition effect of copolymers exceeds that of individual PVCap or PVP, providing a new pathway for developing more effective vinyl lactam polymer inhibitors.

### 3.2. Amide Polymer Inhibitors

Amide polymers, such as polyvinylamide, polyalkylacrylamide, and polyacrylamide [118], including polyIPMA, polyIPAm, and polyvinylacrylamide [119], play significant roles in hydrate inhibition. These polymers, particularly acrylamide polymers, exhibit excellent hydrate inhibition performance due to their structural characteristics [120]. For example, polyIPMA with a bimodal molecular weight distribution can effectively prevent hydrate formation at a concentration of 0.5 wt% [121], maintaining high supercooling conditions (24.1 K) for 20 h. Further research shows that although polyVIMA, made from N-methyl-N-vinylacetamide (VIMA), has weak inhibitory effects alone, it shows better inhibition when copolymerized with compounds like isopropyl methacrylamide [122]. Polymers based on VIMA synthesized by Exxon [57], such as vinyl butyrate polymers, also perform well. Homopolymers or copolymers containing isopropylacrylamide, isopropyl methacrylamide, and dimethyl hydrazine methacrylamide have potential in amide-based KHIs. The KHI effect is generally related to the methyl content in the polymer backbone, with methyl-containing polymers showing superior hydrate inhibition. This finding emphasizes that fine-tuning and optimizing polymer structures, such as introducing methyl groups and adopting suitable copolymerization strategies, can significantly enhance inhibitory effects.

### 3.3. Natural Green Inhibitors

Despite the significant effectiveness of KHIs in reducing hydrate formation and their much lower dosage requirements compared to thermodynamic inhibitors, they often pose challenges related to biodegradability and biotoxicity. To address these issues, researchers have begun exploring natural products as KHIs, seeking effective and environmentally friendly solutions. Natural products such as antifreeze proteins (AFPs), amino acids, pectin, and waterborne polyurea/polyurethane (WPUUs) have shown remarkable inhibitory capabilities. Notably, AFPs outperform the commercial inhibitor PVP in inhibiting hydrate formation [123]. Amino acids, pectin, and certain types of starch, such as cassava starch, have also proven effective in inhibiting hydrate formation [124,125,126,127,128]. These natural products prevent hydrate formation by disrupting the hydrate cage structure and forming hydrogen bonds with water molecules. Short peptides rich in alanine and specific amino acids such as glycine, alanine, l-serine, and l-proline are considered promising green inhibitors [129]. In summary, using natural products as hydrate inhibitors not only provides an effective strategy to reduce environmental pollution and address the biodegradability and toxicity issues of KHIs but also guides the development of a new generation of green hydrate inhibitors. The application of these natural products demonstrates excellent inhibitory performance and emphasizes the importance of being environmentally friendly and non-toxic. This provides critical scientific and technological support for the greening and sustainable development of hydrate management in the energy sector.

### 3.4. Ionic-Liquid-Based Inhibitors

Recent studies have shown that ionic liquids, particularly imidazolium-based ones, can serve as dual-functional hydrate inhibitors [130]. These studies suggest that for ionic liquids to effectively inhibit hydrate formation, the cation should have a shorter alkyl chain, while the anion should possess strong hydrogen-bonding capabilities [131,132]. Conversely, ionic liquids with longer alkyl chains may provide better kinetic inhibition by more effectively adsorbing onto the hydrate crystal surface [133]. However, imidazolium-based ionic liquids have not demonstrated significant hydrate inhibition capabilities compared to traditional inhibitors [134,135], prompting researchers to explore other types, such as pyridinium [136,137], ammonium [138,139,140], and phosphonium-based ionic liquids [141]. In this exploration, ammonium-based ionic liquids [142] like tetramethylammonium chloride (TMACl) [143] and tetraethylammonium chloride (TEACl) [144] have shown inhibitory effects on hydrate formation, with some demonstrating potential as kinetic inhibitors. These findings highlight the importance of the cation alkyl chain length and the hydrogen-bonding ability of the anion in enhancing hydrate inhibition efficiency. In summary, research on ionic liquids as hydrate inhibitors has revealed that carefully selecting cation and anion structures can effectively inhibit hydrate formation and growth. Although the inhibition effects of imidazolium-based ionic liquids were not as anticipated, exploring ammonium-based and other types of ionic liquids offers new directions for developing novel, efficient hydrate inhibitors. Future research will need to delve into the mechanisms of ionic liquids and optimize their structures to achieve higher inhibition efficiency.

## 4. Mechanism of Inhibition of Hydrate Generation by Kinetic Inhibitors of Natural Gas Hydrates

### 4.1. Perturbation Suppression Mechanism

**Figure 11:**
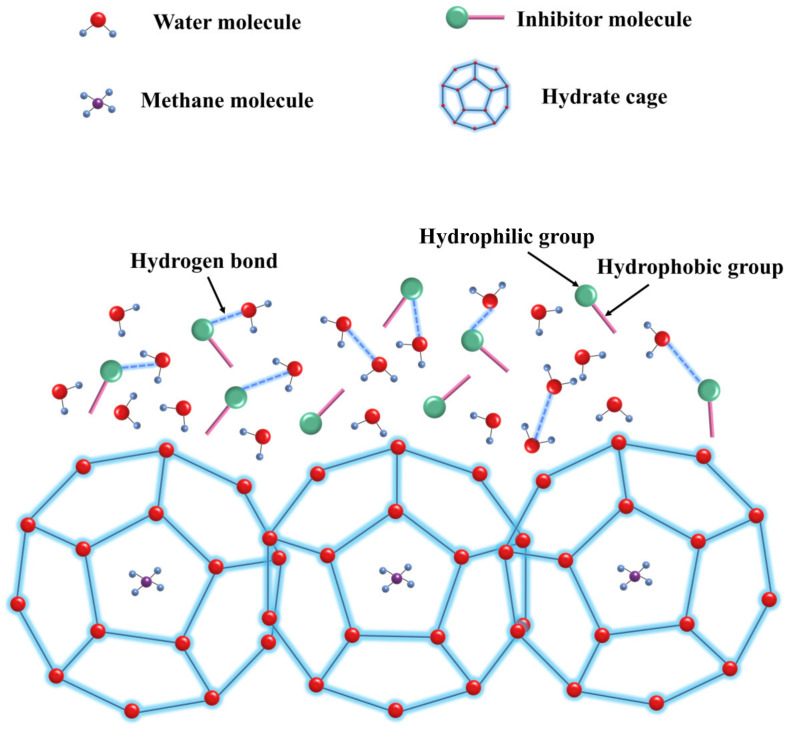
Schematic diagram of perturbation suppression mechanism [102].

Hydrate inhibitors effectively prevent the formation and growth of hydrates through specific mechanisms. They form hydrogen bonds with water molecules, disrupting their orderly structure and, thus, preventing the formation of hydrate cage structures before nucleation [145]. After nucleation, the inhibitor molecules continue to adsorb onto the surface of hydrate crystals, blocking further crystal growth [146,147,148,149], as shown in Figure 11.

**Figure 12:**
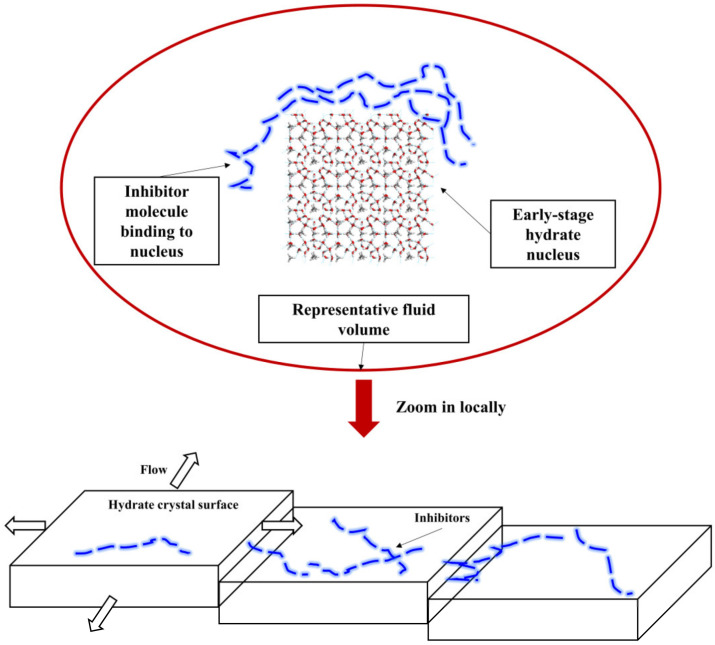
Conceptual model for inhibitor binding and crystal growth inhibition [96].

Anderson et al. [96]. utilized molecular simulation techniques to examine the performance of inhibitors such as PVCap, PVP, PEO, and VIMA, finding that the inhibitory effect is positively correlated with the binding energy between the inhibitor and the hydrate crystal surface. They found that higher binding energy results in better inhibitory effects, with the performance ranking as VIMA > PVCap > PVP > PEO. Inhibitors with strong binding energies form robust hydrogen bonds with the hydrate crystal surface due to their specific charge distribution and size, as shown in Figure 12. Unlike polymers such as PVP and PVCap, substances like amino acids inhibit the nucleation and growth of hydrates by disrupting the orderly structure of water molecules, demonstrating diverse inhibition mechanisms [150]. Lederhos et al. [39]. suggest that the molecular structures of PVP and PVCap contain five-membered and seven-membered lactam rings, similar in size to the pentagonal and hexagonal structures in hydrate cages. When these rings adsorb onto hydrate grains through hydrogen bonds, they create steric hindrance and inhibit grain growth. High molecular side chain groups enter the cavities of the hydrate cages, adsorbing onto specific surfaces of the growing hydrate crystals, and form hydrogen bonds with the hydrate surface, thereby preventing the hydrate from reaching the thermodynamic conditions favorable for its growth to a critical size.

### 4.2. Layer Mass Transfer Obstruction Mechanism

**Figure 13:**
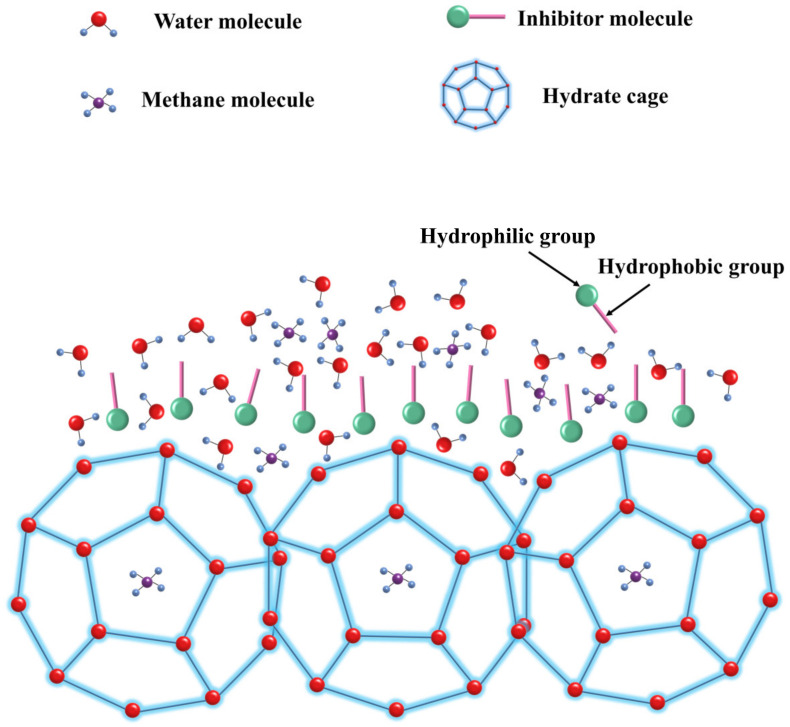
Schematic diagram of layer mass transfer obstruction mechanism [96,152].

When the mass fraction of inhibitors is sufficiently high, the polymer layer formed at the gas–liquid interface can significantly hinder the movement of water and guest molecules, increasing mass transfer resistance and effectively inhibiting hydrate growth. This mechanism is emphasized by Kuznetsova et al.’s [151] layer mass transfer resistance hypothesis, which highlights the role of inhibitors aggregating at the gas–liquid interface to form a polymer layer that increases mass transfer resistance, as shown in Figure 13.

Application studies using molecular simulation technology have found that inhibitors such as PVP tend to concentrate at the gas–liquid interface [153], forming hydrogen bonds with interfacial water molecules and preventing them from participating in the formation of hydrate cage structures. Similarly, inhibitors such as PVCap and PVP form polymer layers on the hydrate crystal surface, significantly increasing mass transfer resistance at the gas–liquid interface, slowing down molecular diffusion, and, thus, reducing hydrate growth rates [154]. An increase in polymer layer thickness further slows hydrate growth, demonstrating the significant role of the polymer layer in preventing hydrate growth. This study reveals a new mechanism where inhibitors form polymer layers at the gas–liquid interface, effectively inhibiting hydrate nucleation and growth by increasing intermolecular mass transfer resistance [69,155,156]. Kvamme’s research indicates that after hydrate nucleation, kinetic factors dominate its growth. Inhibitors like PVP form a layer between water and guest molecules, effectively increasing the mass transfer resistance between methane and water molecules, thereby inhibiting further hydrate growth. This phenomenon is known as the layer mass transfer resistance effect. Molecular dynamics simulations confirm that PVP tends to form hydrogen bonds with water molecules on the hydrate surface, effectively preventing hydrate growth [157]. The study also found that PVCap interacts better with hydrate solutions than PVP, making it a more suitable choice for kinetic inhibitors. Although PVCap is less soluble in water than PVP, adding hydroxyl groups to its monomer ring can significantly enhance its hydrophilicity and water solubility, improving its adhesion to the hydrate surface. VC-713 also showed good interaction with hydrates [158].

### 4.3. Adsorption and Spatial Obstruction Mechanism

**Figure 14:**
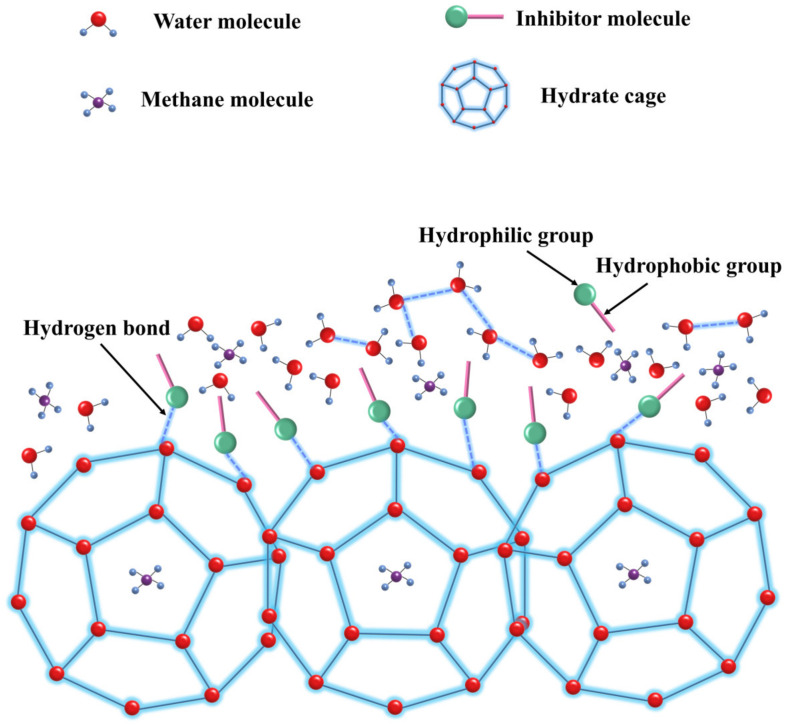
Schematic diagram of adsorption and spatial obstruction mechanism [151,152].

The surface adsorption inhibition hypothesis is currently the most widely accepted theory for understanding the mechanism of kinetic inhibitors. It emphasizes that inhibitors prevent further hydrate growth through hydrogen bonding on the hydrate crystal surface [39], as shown in Figure 14. The addition of inhibitors can not only alter the growth faces of hydrate crystals but also, at appropriate concentrations, cause the cessation of crystal growth. This effect is attributed to the irreversible adsorption of inhibitor molecules on the hydrate surface and their ability to cover the crystal surface, preventing the crystal from reaching the critical growth size [159].

The chemical structure of inhibitors, especially the hydrogen bonds formed between their hydrophilic functional groups and water molecules, and the incomplete cage-like guest molecules simulated by structures such as side-chain alkyls [160,161] collectively increase the difficulty of hydrate crystal growth. Techniques like small-angle neutron scattering support this surface adsorption mechanism, revealing a direct correlation between the increase in growth-impeding sites and the enhancement of inhibitory effects [162]. Further research has found that different types of inhibitors, such as PVCap and VP/BA, exhibit varying efficiencies in inhibiting the nucleation and growth of hydrates, indicating that the chemical structure of polymers significantly affects their inhibitory effects. Kinetic inhibitors inhibit hydrate growth by forming an adsorption layer on the hydrate crystal surface, and their efficiency depends on the concentration of the inhibitor, its chemical structure, and its interaction with water molecules [163].

PVP and PVCap, as polymer inhibitors, primarily use an adsorption mechanism to prevent hydrate growth. However, these two inhibitors exhibit significant differences in their inhibition performance and adsorption behavior. PVCap shows superior inhibition performance compared to PVP, mainly because PVCap can form a denser adsorption layer on the nucleation surface, while the adsorption layer formed by PVP is relatively looser [164]. The effectiveness of hydrate inhibition relies on three key factors: higher adsorption capacity, a rigid adsorption layer, and strong affinity with the nucleation surface [165]. PVCap excels in these aspects, as its dense adsorption layer is more challenging for guest molecules and water molecules to penetrate, effectively inhibiting the nucleation and growth of hydrates. The memory effect of hydrates indicates that the dense adsorption film formed by PVCap is easier to wash away than the loose film formed by PVP, making PVP more effective in eliminating this memory effect. Experiments demonstrate that under the same conditions, a larger proportion of the PVCap film can be cleaned off, whereas the cleaning rate of the PVP film is lower. PVCap and PVP demonstrate different adsorption behaviors on hydrate surfaces. PVCap tends to follow the BET adsorption model, whereas PVP exhibits the Langmuir adsorption model. This difference may impact their adsorption efficiency and ability to inhibit hydrate formation [36]. The polymer’s affinity for the hydrate surface is related to the free binding energy of the adsorption monomer and the number of structural units on the hydrate surface, which, in turn, influences the inhibition effect [28].

The effectiveness of kinetic inhibitors is primarily due to their dual adsorption roles during the nucleation and growth processes of hydrates. This includes their adsorption on non-hydrate structures, which prevents the formation of hydrate cage structures during nucleation, especially on impurities or surfaces within the system, such as oxides of Si and Fe and hydrophobic container surfaces. Additionally, their adsorption on hydrate structures or crystals directly disrupts the nucleation process, preventing further growth [164,166]. Molecular simulation studies have detailed the interactions between kinetic inhibitors, such as PVP, and hydrate surfaces, showing that specific groups (e.g., oxygen on the pyrrolidone ring) form hydrogen bonds with the hydrate surface, effectively preventing further growth. The adsorption of inhibitors not only deforms hydrate crystals and isolates active centers on the crystal surface, creating spatial hindrance, but also affects the formation process [167]. Furthermore, interactions between inhibitors and methane molecules prevent methane from entering and filling the hydrate cavities, thus inhibiting hydrate formation [168].

### 4.4. Particle Adhesion Mechanism

The aggregation of hydrate particles is a key factor contributing to pipeline blockages [169,170], particularly during the growth and aggregation stages following hydrate nucleation. The adhesion between particles facilitates the aggregation of hydrate particles, thereby increasing the risk of blockages. Kinetic inhibitors can significantly modify the physical and chemical properties of hydrate particles through interactions with the hydrate surface or cages. These modifications adjust the adhesion forces between particles, thereby reducing their tendency to aggregate.

**Figure 15:**
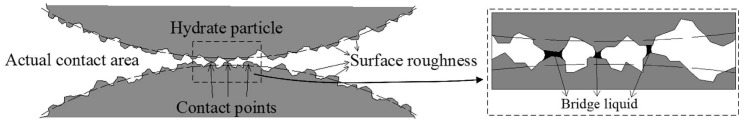
Surface contact geometry of particle–particle adhesion and magnified liquid bridge [179].

Research indicates that the primary causes of hydrate blockage are the capillary forces resulting from liquid bridge formation between particles and the wettability of these particles [171,172,173]. Oleophilic hydrate particles show a reduced risk of aggregation, significantly influenced by the surface wettability and interactions between particles [174,175,176,177]. Additionally, hydrate particle aggregation is driven not only by the adhesion force between particles but also by the transformation of water droplets into hydrates upon contact with particles, thereby bonding them together [178]. Using anti-agglomerates such as PVA can alter the morphology of hydrate particles, increasing their surface roughness, reducing the contact area and adhesion force between particles, and, thus, effectively preventing aggregation [179], as shown in Figure 15.

### 4.5. Other Mechanism

Moon [180] was the first to propose the interfacial energy efficiency mechanism, which explains how PVP increases interfacial energy to affect the nucleation process of hydrates, thereby extending the time required to form larger critical clusters. Simulation studies further revealed PVP’s role at the hydrate interface, demonstrating that PVP maintains a certain distance from the hydrate surface. This suggests that hydrate crystals still have room to grow even in the presence of PVP. PVP’s intervention disrupts the orderly state of water molecules, slowing down their aggregation into clusters and, thus, affecting the nucleation rate. Although PVP effectively inhibits the nucleation process of hydrates, it shows limitations in preventing further growth once a nucleus has formed. This indicates that PVP’s inhibitory effect is primarily concentrated in the initial stage of hydrate formation, specifically the nucleation process.

The interfacial energy efficiency mechanism offers valuable insight into how PVP prolongs the hydrate nucleation process by influencing interfacial energy. While the interaction between PVP and the hydrate interface does not completely inhibit crystal growth, its disruption of the orderly arrangement of water molecules effectively slows the nucleation rate. Consequently, PVP’s inhibitory effect is primarily restricted to the nucleation stage of hydrate formation, with minimal impact on the growth of already-formed hydrate nuclei.

Yagasaki [181] conducted an in-depth study using molecular dynamics to investigate the interaction mechanisms between guest molecules and inhibitor molecules on the hydrate surface. Analysis of the free energy profiles of various guest molecules revealed that spherical molecules with a diameter of approximately 0.5 nm have the highest affinity for the hydrate surface. However, this high affinity does not directly affect the macroscopic mass transfer process of guest molecules during the growth of gas hydrate crystals.

For inhibitor molecules, especially PVCap, the mechanism of action primarily involves the formation of hydrogen bonds between the amide functional groups and the water molecules on the hydrate surface, promoting the adsorption of PVCap onto the hydrate surface [160]. This hydrogen bonding is the main driving force for PVCap’s adsorption, although it does not directly influence surface affinity. Additionally, the adsorption affinity on the hydrate surface is affected by the side chain distance and the shape of the hydrate cages. The adsorption affinity of hydrophobic functional groups in kinetic inhibitors is independent and does not interact with each other. Importantly, whether the molecules adsorbed on the hydrate surface are inhibitor molecules or spherical guest molecules is primarily determined by the entropy stabilization of the surface cavities of the hydrate [181,182]. This finding provides a crucial perspective for understanding the inhibition mechanism of hydrate formation.

## 5. Conclusions and Outlook

In summary, the study of kinetic inhibitors for natural gas hydrates demonstrates significant potential in addressing challenges such as pipeline blockages caused by hydrate formation. Through a thorough analysis of the development history, chemical properties, mechanisms of action, and practical application efficiency of various kinetic inhibitors, this paper identifies several key development directions and future research priorities.

Firstly, current research highlights the importance of continuing to explore new kinetic inhibitors, particularly those compounds with low environmental impact and high efficiency. With increasing environmental standards, it has become crucial to identify and develop biodegradable inhibitors that have minimal ecological impact. Natural-source inhibitors, such as antifreeze proteins and specific amino acids, show potential as efficient and environmentally friendly kinetic inhibitors. Secondly, a deep understanding of the mechanisms of action of kinetic inhibitors is essential for designing more effective compounds. Further research is needed on the molecular-level interactions and the relationship between the structural characteristics of inhibitor molecules and the hydrate formation process. Specifically, exploring the interactions between inhibitors and hydrates through molecular simulations and advanced experimental techniques will help optimize the molecular design for more effective hydrate formation control. Additionally, given the complexity of hydrate formation, a single inhibitor may not meet all application scenarios. Therefore, developing multifunctional inhibitor systems that combine kinetic inhibition and thermodynamic stability will be a key direction for future research. Such systems could leverage the complementary strengths of different inhibition mechanisms to achieve more robust and versatile hydrate management solutions. Finally, strengthening the link between laboratory research and field applications by validating the effectiveness and feasibility of kinetic inhibitors through real-world oil and gas field applications is crucial for promoting the widespread commercialization of kinetic inhibitors. Field trials and long-term studies in diverse operational conditions will be essential to demonstrate the practical benefits and address any potential challenges in large-scale deployment.

Research on kinetic inhibitors for natural gas hydrates is rapidly advancing, presenting both significant challenges and opportunities. Continuous scientific exploration and technological innovation will undoubtedly lead to the development of more efficient and environmentally friendly kinetic inhibitors, providing critical support for the safe and efficient extraction of natural gas hydrates.

## Figures and Tables

**Table 1:** Three phases of kinetic hydrate inhibitor research.

Time	Research Phase	Thrust	Representative Inhibitors
1991–1995	Phase I	Discovery of kinetic inhibitors and screening	Polyvinylpyrrolidone (PVP)
1995–1999	Phase II	Improvements based on studies of first-generation kinetic inhibitors	Polyvinyl caprolactam (PVCap) and PVCap copolymer (PVPNC)
1999–present	Phase III	Design of kinetic inhibitor molecules using molecular simulation methods to develop kinetic inhibitors with improved performance	Vinyl lactam-based polymer inhibitors, ionic liquid-based inhibitors, and complex inhibitors

## Data Availability

No new data were created or analyzed in this study.
